# A Rare Case of Bacillus cereus Septic Arthritis

**DOI:** 10.7759/cureus.33148

**Published:** 2022-12-30

**Authors:** Peter Newman, Monica Garcia, Rebecca Ward, John Killian, Sharon Mayberry

**Affiliations:** 1 Pediatric Residency, University of Florida College of Medicine, Pensacola, USA; 2 Pediatric Hospitalist, University of Florida College of Medicine, Pensacola, USA; 3 Pediatric Orthopedics, Studer Family Children's Hospital at Ascension Sacred Heart, Pensacola, USA

**Keywords:** penetrating wounds, knee swelling, joint swelling, bacillus cerrus, septic arthritis

## Abstract

Joint infections are rare in pediatrics, and those caused by *Bacillus cereus* are practically unheard of. In this case report, we examine a singular case of *B. cereus* septic arthritis in a child, with the purpose of educating clinicians on the presentation, inpatient and outpatient management, and clinical outcome. We report the case of a previously healthy pediatric patient who presented to the emergency department with symptoms of septic arthritis of the left knee. The orthopedics team performed arthroscopy and debridement, and the synovial fluid culture grew *B. cereus*. To our knowledge, this is the first case report on septic arthritis caused by *B. cereus* in the pediatric population. The treatment protocol consisted of intravenous vancomycin for one week, followed by three weeks of oral ciprofloxacin therapy. The patient had an excellent clinical outcome and returned to normal mobility without limitations. Despite being ubiquitous in the environment, extra-intestinal *B. cereus* infection is exceedingly rare in immunocompetent individuals. It is so rare, in fact, that it is often dismissed as a lab contaminant. In this case, we demonstrated that cooperation between multiple disciplines offers good clinical outcomes for rare infections, especially those in pediatrics.

## Introduction

Joint infections are rare in pediatrics, and those caused by *Bacillus cereus* are practically unheard of. *Bacillus cereus* is a gram-positive, anaerobic bacteria often found in the soil, and on vegetation [1]. It is most remembered for the contamination of rice after outbreaks on multiple occasions in which consumed Chinese fried rice was contaminated with spores [2]. Most commonly, *B. cereus* exposure presents with either upper gastrointestinal (i.e., nausea, vomiting) or lower gastrointestinal complaints (i.e., abdominal pain, diarrhea), but can have elements of both. Despite being ubiquitous in the environment, extra-intestinal *B. cereus* infection is exceedingly rare in immunocompetent individuals. It is so rare, in fact, that it is often dismissed as a lab contaminant [3]. 

## Case presentation

A previously healthy seven-year-old male, fully immunized, presented on two separate occasions to the pediatric emergency department after falling onto his left knee while running around the home. The mother stated the patient was running around the house when he tripped and hit his left knee on a wall. She later noted that the wall on which he hit his knee had wood-finishing nails protruding from it. He was able to ambulate after the injury with only slight bleeding from the wound. Three hours later, she found him crying and complaining of knee pain. His mother noticed the knee was very swollen, and he was unable to move it without causing severe pain. 

His mother took him to one of our free-standing ER about five to six hours later. The initial exam showed a moderately erythematous and swollen left knee with an abrasion. An x-ray of the left knee demonstrated no fractures or effusions, so his knee was wrapped with an ace bandage, and he was sent home. Over the next several hours, his pain continued to worsen; he became inconsolable and unable to sleep. His mother took him to our main hospital ED.

**Figure 1:**
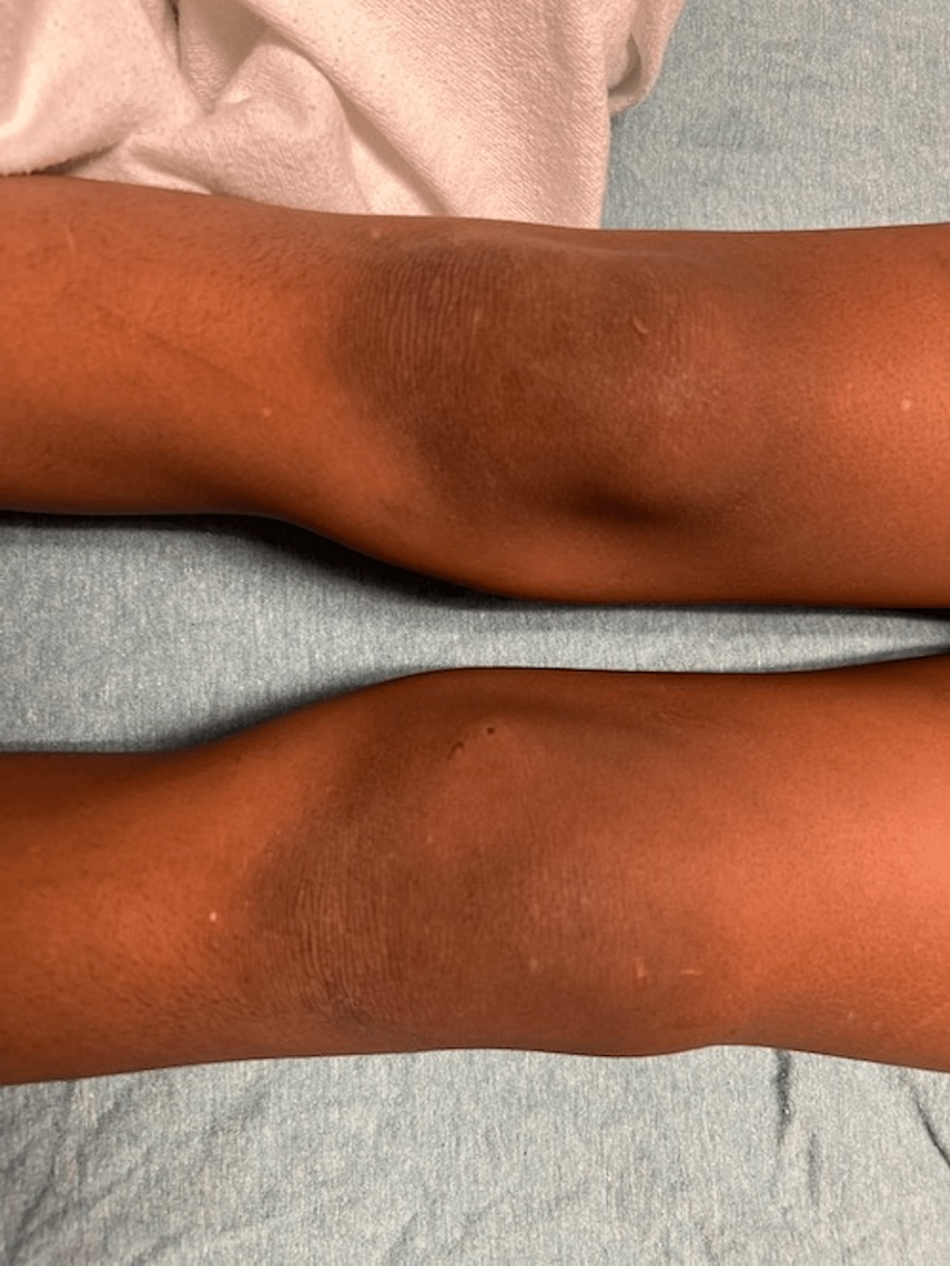
Anterior view of the left knee. Anterior view of the left knee (the knee closest to the bottom of the picture) compared to the right knee showing swelling and a puncture wound on the medial aspect.

**Figure 2:**
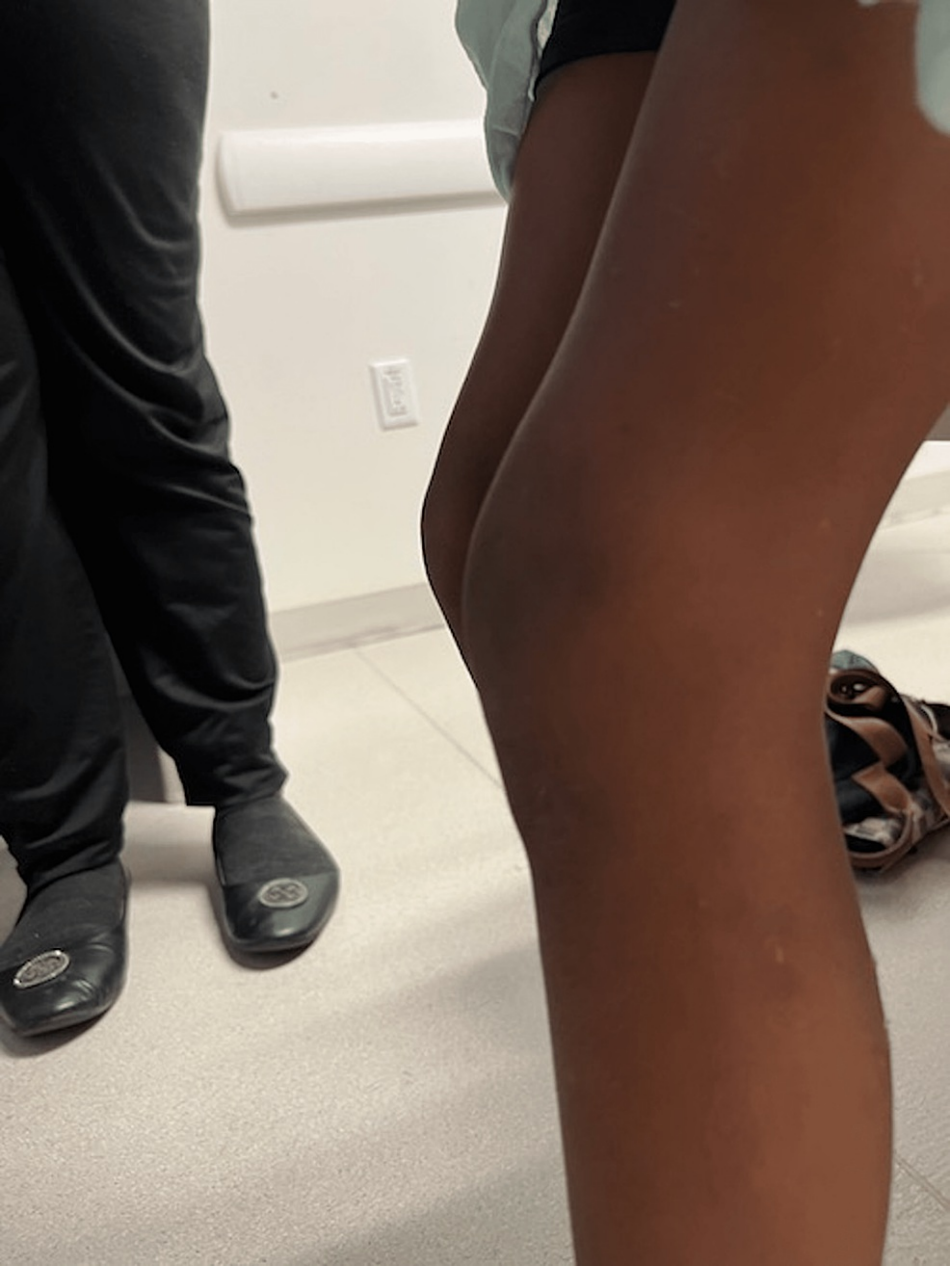
Lateral view of the left knee. Lateral view of the left knee swelling compared to the right knee (furthest from the image).

**Figure 3:**
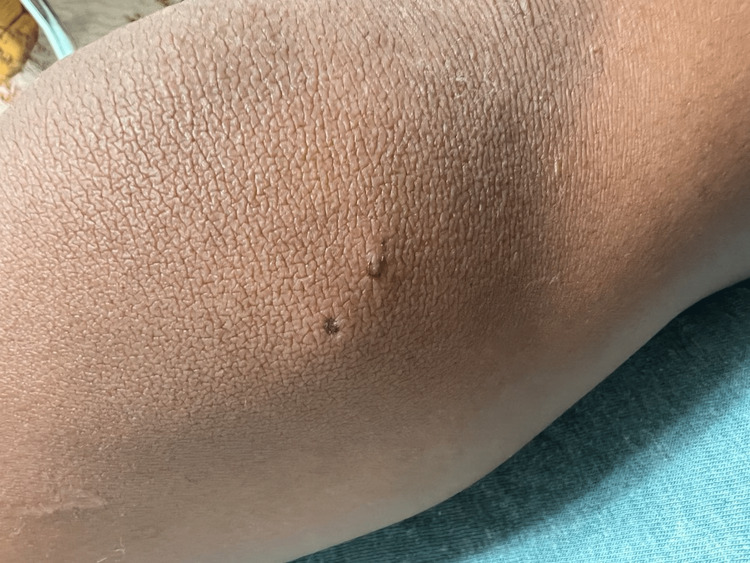
Medial view of the left knee. Medial view of the left knee puncture wound.

During this second visit, the knee was diffusely swollen with two puncture wounds on the medial aspect of the left knee with a limited range of motion due to pain with moderate tenderness over the puncture wounds (Figures 1-3). He was unable to bear weight.

**Figure 4:**
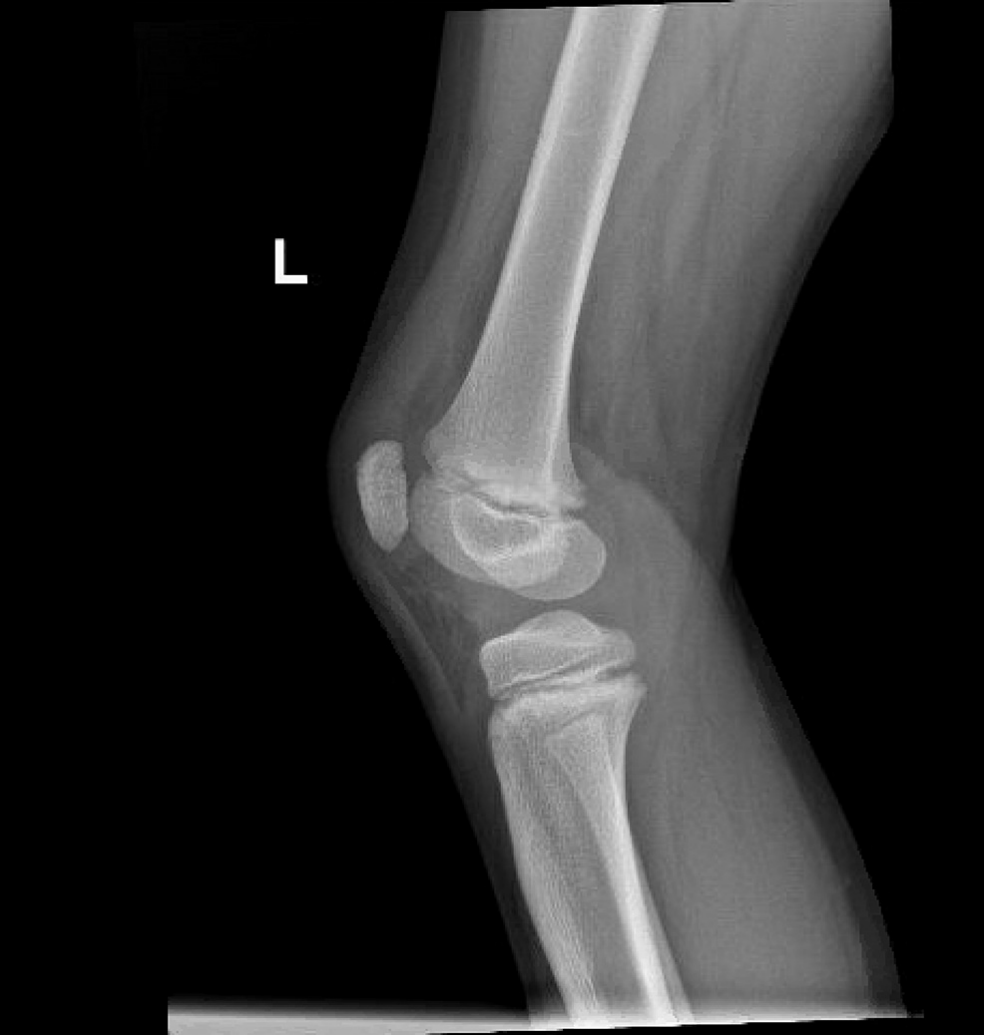
Lateral x-ray view of the left knee.

**Figure 5:**
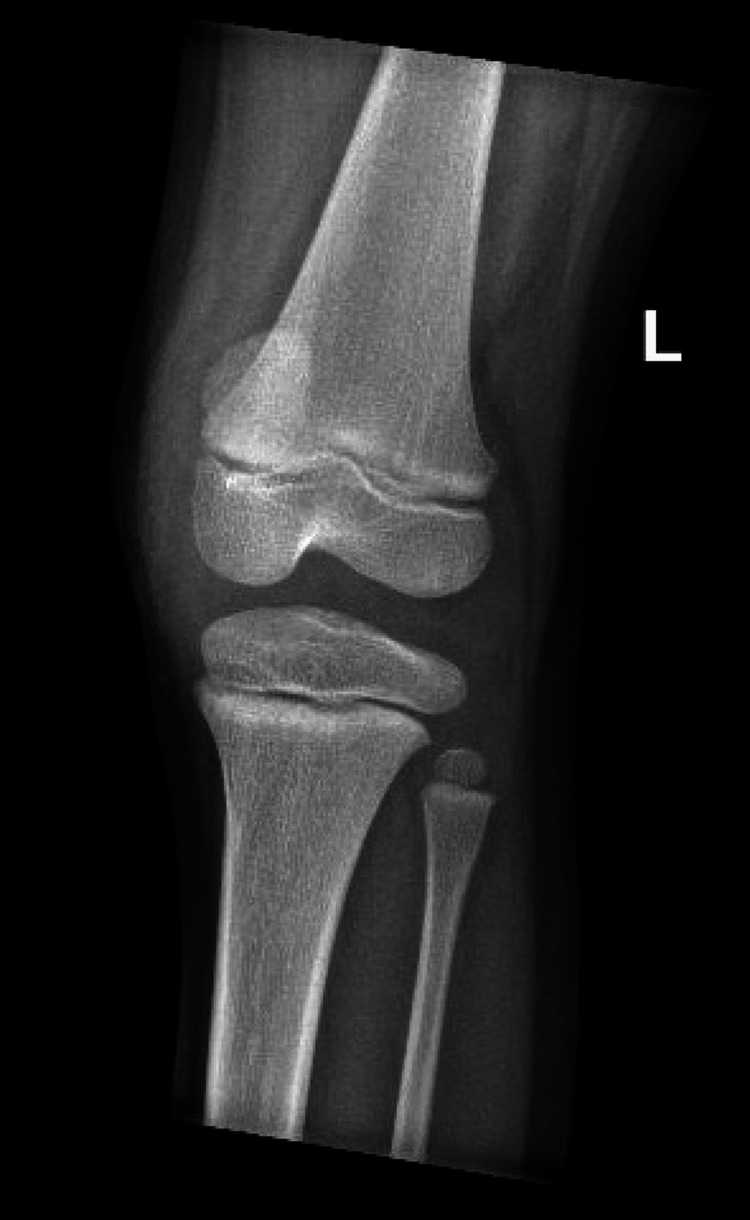
Posterior x-ray view of the left knee.

**Figure 6:**
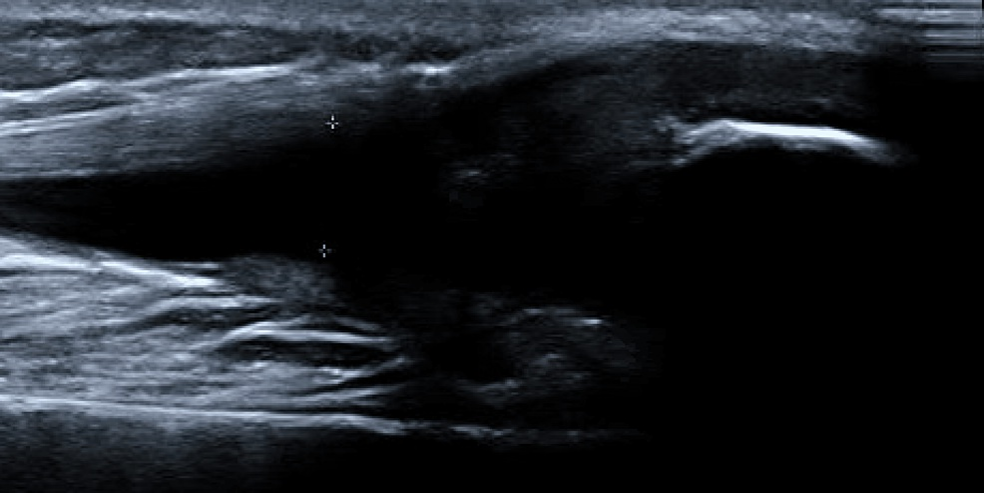
Long segmented view of the left knee US. Long segmented view of the left knee US with the pocket of fluid near the patella, marked by plus signs. US: ultrasound.

Due to concerns about his rapidly worsening condition, he had laboratory blood work, an x-ray of the knee, and an ultrasound (US) of the joint. Laboratory blood tests were significant for an elevated white count with a neutrophil predominance with normal inflammatory markers (Table 1). The repeat complete knee x-ray (Figures 4, 5) and the US of the knee (Figure 6) were concerning for a Salter-Harris type 1 fracture of the distal femur and an irregularity of the cortex of the upper third of the left patella, indicating a possible incomplete impaction fracture, along with a joint effusion with no synovial thickening. At this time, pediatric orthopedics was consulted, who, after evaluation, took the patient for an immediate arthroscopy and aspiration with irrigation and debridement of the left knee joint, and the patient was admitted to their service. 

**Table 1:** Laboratory results. Inpatient and outpatient labs. ESR: erythrocyte sedimentation rate, CRP: C-reactive protein.

Initial labs		
Complete blood count	Result	Normal range
WBC	13.2 k/uL	4-11 k/uL
Hgb	12.4 g/dL	13.7-17 g/dL
Hct	38.70%	40-50%
Platelet	315 k/uL	150-400 k/uL
Neutrophil count	71.70%	
Absolute neutrophils	9.49 k/uL	1.6-8.5 k/uL
Absolute monophils	1 k/uL	0.03-0.6 k/uL
Inflammatory markers		
ESR	6 mm/h	<10 mm/h
CRP	0.25 mg/dl	<0.9 mg/dl
Repeat labs		
Complete blood count	Result	Normal range
WBC	7.6 k/uL	4-11 k/uL
Hgb	10.2 g/dL	13.7-17 g/dL
Hct	31.70%	40-50%
Platelet	277 k/uL	150-400 k/uL
Neutrophil count	43.90%	
Absolute neutrophils	3.33 k/uL	1.6-8.5 k/uL
Absolute monophils	0.89 k/uL	0.03-0.6 k/uL
Repeat inpatient inflammatory markers		
ESR	16 mm/h	<10 mm/h
	14 mm/h	
	14 mm/h	
	12 mm/h	
CRP	5.79 mg/dl	<0.9 mg/dl
	1.63 mg/dl	
	1.08 mg/dl	
	0.46 mg/dl	
Repeat outpatient inflammatory markers		
ESR	9 mm/h	<10 mm/h
CRP	0.05 mg/dl	<0.9 mg/dl
	0.03 mg/dl	

**Figure 7:**
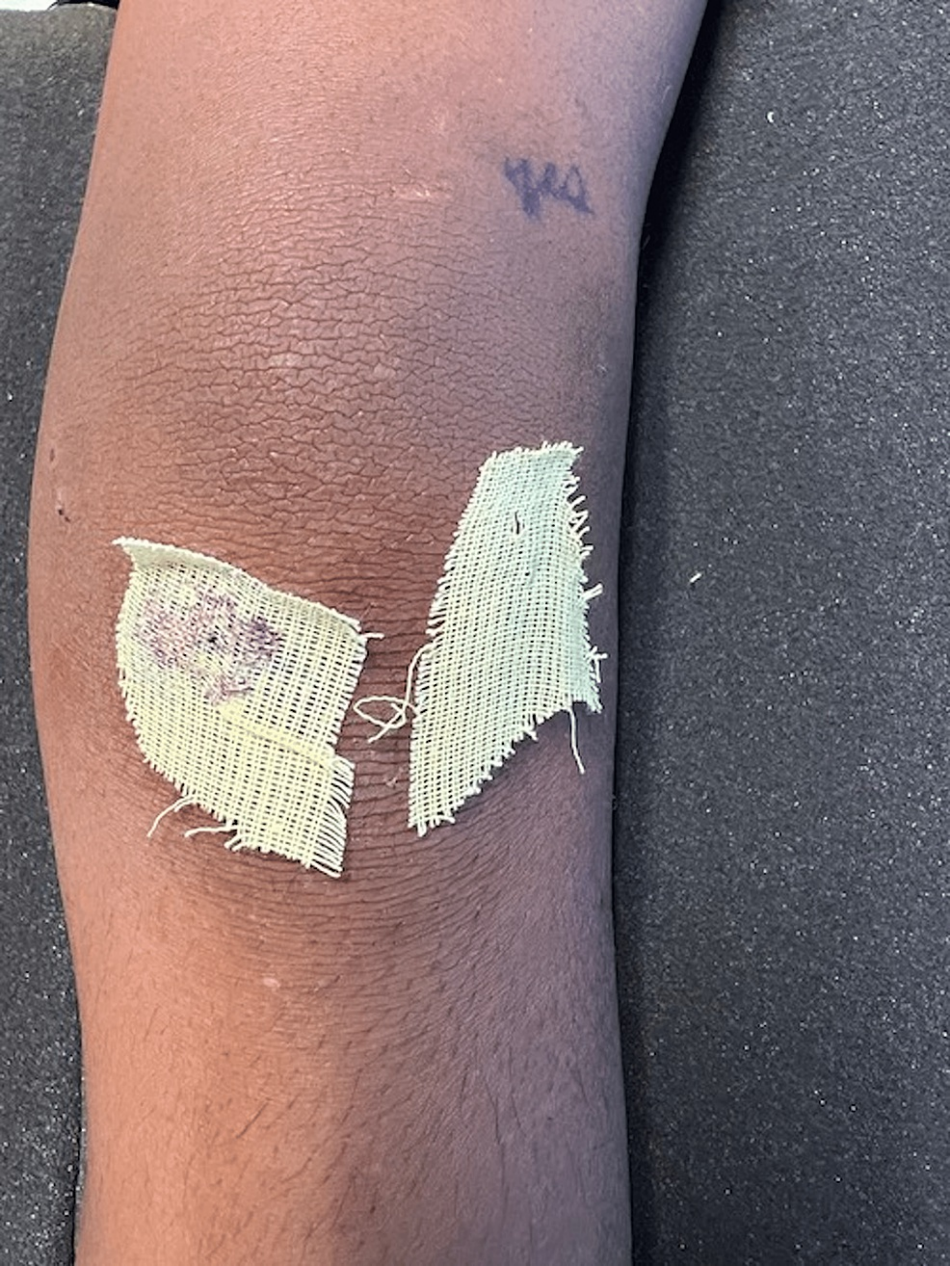
Anterior view of the left knee post-operative day one.

**Figure 8:**
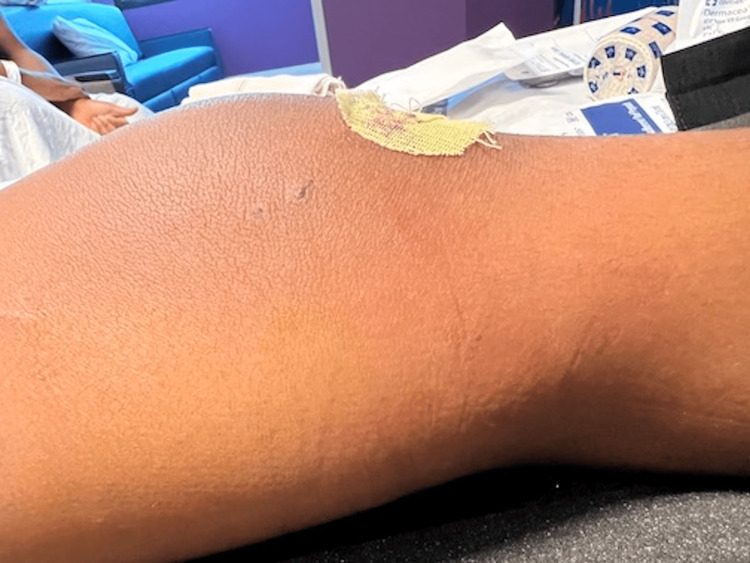
Medial view of the left knee post-operative day one.

Before arthroscopy and aspiration with pediatric orthopedics, the differential diagnosis included cellulitis, deep subcutaneous abscess, septic arthritis, and osteomyelitis. During the procedure, 20 mL of cloudy fluid was aspirated from the knee joint and sent for culture, along with blood and surgical wound cultures. During the arthroscopy, the knee was noted to have a diffuse injection of the synovium, along with mild hemarthrosis with clots of blood present. No other obvious injury to the area or the bone of the distal femur was noted. He had complete irrigation and debridement of the knee joint. His left knee was dressed and placed in a knee immobilizer (Figures 7, 8). 

Following the procedure, he was empirically started on intravenous cefazolin. After 24 hours, the patient’s synovial fluid culture grew *Bacillus cereus*. At this time, patient care was transferred to the pediatric hospitalist service for further infection management of left knee septic arthritis.

We discussed with the patient’s mother the unusual nature of her child’s infection and the more typical intestinal infections that *Bacillus cereus* is known to cause. After this explanation, the mother recollects that a day or so before his fall, the patient had reheated fried rice hibachi for dinner, which was also fed to their dog, who may have licked the patient's wound following the injury. 

**Figure 9:**
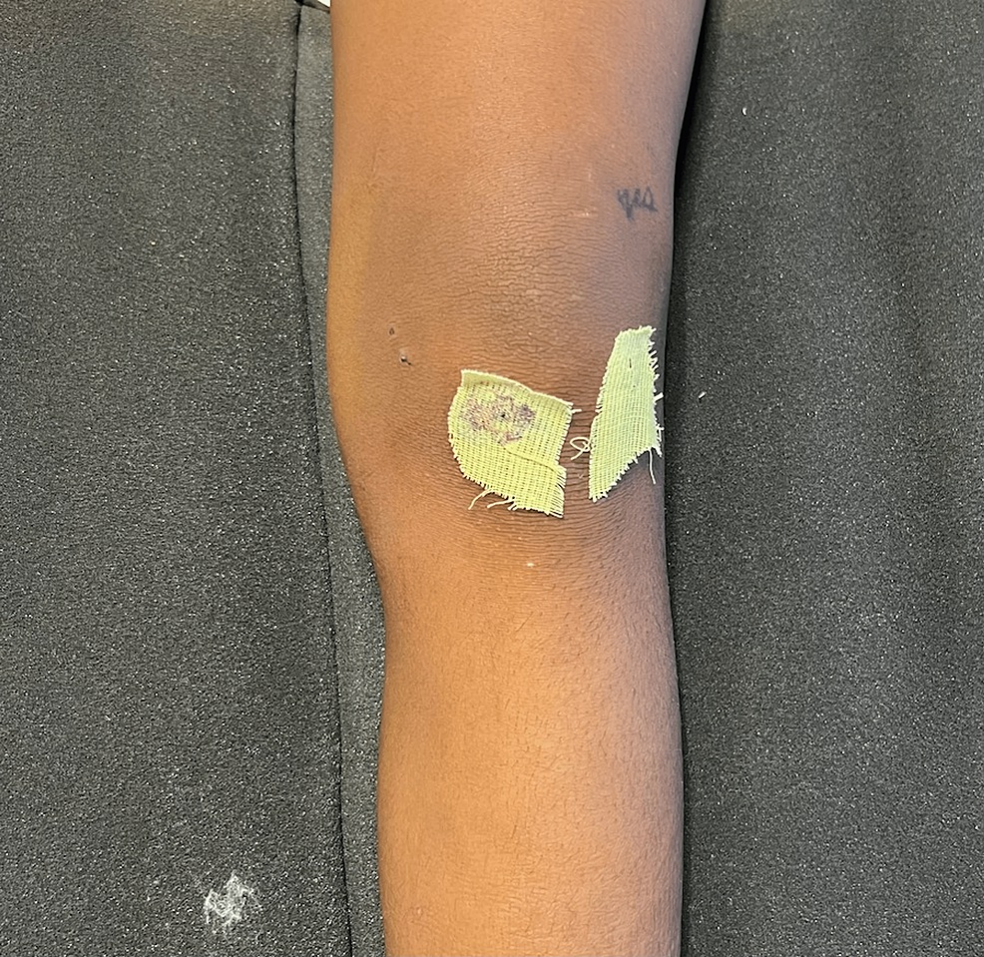
Anterior view of the left knee post-op day seven.

**Figure 10:**
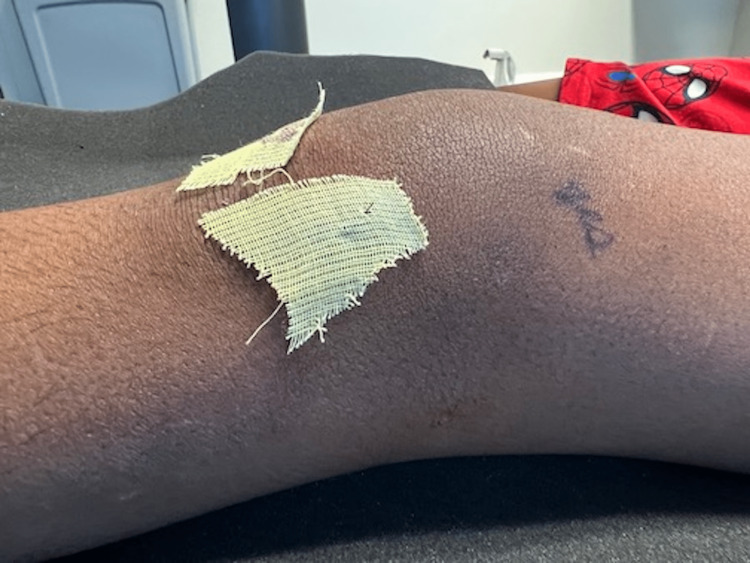
Lateral view of the left knee post-op day seven.

While in the hospital, the patient's peripheral venous line was changed from IV; the reasoning for changing cefazolin to vancomycin was the added antibiotic sensitivity resulting from background research on penetrating trauma with *Bacillus cereus*. It was continued, while in the patient after antibiotic sensitives showed the bacteria to be sensitive to vancomycin, fluoroquinolones, gentamicin, chloramphenicol, erythromycin, rifampin, and tetracyclines. The patient was kept on vancomycin for one week after proper trough levels between 10 mcg/mL and 15 mcg/mL were achieved. While the immediate clinical response was noted, during hospitalization, the response to infection was also monitored with serial blood work (WBC, ESR, and CRP) every three to five days. Renal function was also monitored due to the continued requirement of vancomycin to fight the infection (Table 1). After one week of appropriate vancomycin dosing with clinical and laboratory improvement, the patient was changed to oral ciprofloxacin for an additional three weeks of antibiotic therapy. Throughout hospitalization, the patient’s left knee range of motion, weight-bearing tolerance, and swelling slowly improved (Figures 9, 10), until he ambulated fully without aid, requiring the knee immobilizer until further clearance by orthopedics.

After discharge, he was seen two weeks later with repeat labs that continued to be within the normal range (Table 1). For his four-week follow-up, the patient had a repeat two-view x-ray of the left knee, which showed no obvious evidence of abnormalities around his puncture wound. At this visit, he had completed his antibiotic course, and his repeated CRP continued to be within normal limits (Table 1).

## Discussion

*Bacillus cereus* is a gram-positive, facultatively anaerobic, spore-forming, toxin-creating bacilli that are ubiquitous in the environment. It can often be found in the soil, and on vegetation [1]. It is most commonly associated with rice contamination following multiple outbreaks in which consumed Chinese fried rice was contaminated with spores. Due to the way it is cooked, it is boiled in large quantities and kept unrefrigerated for hours before it is processed (heated or fired) for consumers with rice and other rice-based products and is perhaps best known as a food contaminant. It is during the unrefrigerated stage that the micro-organisms can grow and produce the toxin that is not destroyed by the heating process leading to its infamous way of remembering the organism; “reheated fried rice bacteria” [2]. Most commonly, *Bacillus cereus* exposure presents with either upper gastrointestinal (i.e., nausea, vomiting) or lower gastrointestinal complaints (i.e., abdominal pain, diarrhea), but can have elements of both. Symptoms of this type of *Bacillus cereus* toxin exposure typically resolve within 24 hours of onset [3]. Although rare, the extra-intestinal infection can occur, most commonly as post-traumatic endophthalmitis, but also as bacteremia and endocarditis, post-surgical infection, and can also be associated with prosthetic colonization. The severely immunocompromised, it can result in central nervous system infection [3,4]. In terms of treatment for *Bacillus cereus* infection, GI exposure is typically self-limited, with patients only requiring supportive care. With focal or systemic infections, rapid intervention is required. First-line antibiotics include vancomycin, gentamicin, chloramphenicol, ciprofloxacin, or carbapenems [3,5].

In pediatrics, septic arthritis tends to present with an acutely painful, erythematous joint with fever, and, in those cases involving the lower limb, difficulty with weight bearing can also be seen. The most common causative bacteria found in a pediatric septic joint is *Staphylococcus aureus*, but occasionally it can be caused by bacteria such as *Streptococcus pneumoniae*, *Kingella kingae*, and *Salmonella* species [6,7]. The incidence of septic arthritis in pediatrics is four to five cases per 100,000 people annually, and the most commonly affected are the large joints of the lower limb: the hip, knee, and ankle [7,8]. If a pediatric patient presents with a red, swollen, painful joint and fever, the investigation begins with standard labs, such as complete blood count (CBC), C-reactive protein (CRP), erythrocyte sedimentation rate (ESR), and blood cultures. Ultrasound-guided joint aspiration is also needed to obtain synovial fluid for microscopy and culture. X-rays, while generally not very helpful in the diagnosis of septic arthritis, can reveal any related acute fractures or chronic processes. MRI can also be useful in diagnosing osteomyelitis or abscesses, but it can be expensive and may require sedation in some patients [6-8]. Once the diagnosis has been made, treatment includes two to four weeks of systemic antibiotic therapy, depending on the complexity of the patient and the isolated pathogen. Surgical debridement by arthroscopy of the infected joint may also be needed [8,9].

## Conclusions

To our knowledge, this is the first case report on septic arthritis caused by *B. cereus* in the pediatric population. The treatment protocol consisted of intravenous vancomycin for one week, followed by three weeks of oral ciprofloxacin therapy. Following treatment, he was able to get back to school and ambulate well without any pain or limitations. The knee immobilizer was removed with no restrictions on the range of motion or daily life. 
